# Mechanism of heme oxygenase-1 regulation of ferroptosis in vascular dementia

**DOI:** 10.3389/fnmol.2025.1585079

**Published:** 2025-06-26

**Authors:** Xin-yi Zou, Luo-yang Cai, Jin Zhang, Ying Yuan, Jie Song, Zhao-duan Hu, Xiao-feng Ruan, Rui Peng, Xiao-ming Zhang

**Affiliations:** ^1^College of Acupuncture-Moxibustion and Orthopedics, Hubei University of Chinese Medicine, Wuhan, China; ^2^Hubei Shizhen Laboratory, Wuhan, China; ^3^Sub-Health Institute, Hubei University of Chinese Medicine, Wuhan, China; ^4^Hubei Provincial Hospital of Traditional Chinese Medicine, Wuhan, China

**Keywords:** heme oxygenase 1, ferroptosis, vascular dementia, oxidative stress, lipid peroxidation

## Abstract

Vascular dementia (VaD) is a neurodegenerative disorder characterized by chronic oxygen insufficiency, leading to the generation of oxygen-free radicals, inflammatory responses, disturbances in iron metabolism, lipid peroxidation, and other pathological changes that disrupt intracellular homeostasis. These processes ultimately lead to neuronal death and cognitive dysfunction. Normal neurological functions depend on the capacity of the iron homeostatic system to regulate the balance of oxidative states. Imbalances in iron metabolism render nerve cells highly susceptible to cell death induced by iron accumulation. Ferroptosis is a process in which iron catalyzes the peroxidation of unsaturated fatty acid-rich lipids, with ferrous iron or lipoxygenase acting as catalysts and ultimately resulting in cellular demise. Heme oxygenase-1 (HO-1) is a critical enzyme involved in the cellular response to oxidative stress and is essential for regulating signaling pathways linked to iron-mediated cell death. It protects neuronal cells by mitigating oxidative stress, reducing inflammation, and enhancing mitochondrial function, thereby alleviating cerebrovascular injury and slowing the progression of VaD. This paper provides a theoretical framework for understanding and potentially treating VaD-related neuronal injury through the investigation of ferroptosis mechanisms, the biological functions of HO-1, and its role in regulating ferroptosis.

## 1 Introduction

Vascular dementia (VaD) is a syndrome characterized by cognitive decline resulting from vascular damage to brain tissue, which is caused by chronic hypoperfusion in specific brain regions. It is characterized by memory impairments, cognitive deficits, and a decline in mental activity (Sinha et al., 2020). VaD is ranked as the second most common dementia subtype, following Alzheimer’s disease (AD) (Morgan and Mc, 2024). Epidemiological studies indicate that VaD accounts for 15–20% of dementia cases in Europe and the United States, and up to 30% in developing countries, placing a significant medical and economic burden on societies and families (Wolters and Ikram, 2019). Notably, VaD is distinct among dementia subtypes, as early intervention has shown a potential to reverse cognitive decline (D'Angelo et al., 2020). The primary etiology of VaD is linked to cerebrovascular pathology, leading to chronic cerebral hypoperfusion (CCH), which reduces cerebral blood flow and nutrient supply, while promoting the accumulation of harmful molecules in the brain. This buildup triggers pathophysiological alterations, such as oxidative stress, inflammation, and mitochondrial impairment, which collectively lead to neuronal damage and cognitive deterioration (Wang et al., 2020). Research has shown that elevated iron levels in the brain heighten the likelihood of age-related neurodegenerative disorders (Alrouji et al., 2024). Because the brain has high energy demands, limited energy reserves, and an abundance of phospholipids that serve as substrates for oxidative damage, it is especially vulnerable to disruptions in iron metabolism and the resulting oxidative stress (Altahrawi et al., 2025). This accumulation contributes to the generation of oxidative free radicals and peroxides, which subsequently lead to impaired protein function and ultimately result in ferroptosis (Cobley et al., 2018). Elucidating the relationship between ferroptosis and VaD is crucial, and regulating oxidative stress to mitigate ferroptosis should be explored as a potential avenue for further mechanistic studies on VaD. Heme oxygenase-1 (HO-1), an enzyme critical for combating oxidative stress, is essential for protecting neuronal cells from damage. Emerging evidence suggests that HO-1 exerts a regulatory function in the pathophysiological process of VaD by modulating the ferroptosis pathway (Fu et al., 2023; Wang et al., 2023). However, the exact mechanism by which HO-1 produces its effects remains uncertain. To better clarify the role of HO-1 in VaD, its protective mechanism must be elucidated to inform therapeutic strategies targeting VaD-associated ferroptosis.

## 2 Mechanisms of ferroptosis

### 2.1 Disruptions in iron metabolism

Under physiological conditions, systemic iron homeostasis is maintained through coordinated dietary absorption and macrophage-mediated recycling of senescent erythrocytes (Vogt et al., 2021). Iron is internalized via transferrin–transferrin receptor binding, then reduced and released into the cytoplasm via divalent metal transporter 1 (DMT1). Excess intracellular iron is either sequestered in ferritin or exported through ferroportin to preserve redox balance (Anderson and Frazer, 2017).

Pathological conditions characterized by iron overload or metabolic dysregulation lead to the accumulation of excess ferrous iron (Fe^2+^). In endosomes, ferric iron (Fe^3+^) is reduced to Fe^2+^ by STEAP3 and subsequently transported to lysosomes via DMT1, eventually saturating intracellular iron storage capacity. The Fenton reaction (Fe^2+^ + H₂O₂ → Fe^3+^ + OH^−^ + •OH) generates excessive reactive oxygen species (ROS) that surpass the neutralizing capacity of endogenous antioxidants such as glutathione (GSH) (Long et al., 2020). The role of the Fenton reaction in ferroptosis has highlighted the delicate balance between the physiological necessity of iron and its potential toxicity. In contrast to physiological ROS signaling, pathologically elevated ROS levels are linked to widespread lipid peroxidation and oxidative injury (Zheng et al., 2022). Additionally, nuclear receptor coactivator 4-mediated ferritinophagy promotes the release of redox-active iron into the labile iron pool, thereby facilitating ferroptotic signaling cascades (Li et al., 2024).

### 2.2 Lipid peroxidation

Lipid peroxidation is recognized as a rapid and pivotal event in ferroptosis. Phospholipids, particularly those enriched with polyunsaturated fatty acids (PUFAs) such as arachidonic acid, are highly susceptible to peroxidation in the plasma membrane. This process leads to the disruption of the lipid bilayer, resulting in the generation of cytotoxic by-products—including lipid free radicals, lipid hydroperoxides, and reactive aldehydes—that compromise membrane integrity (Ayala et al., 2014). PUFAs are critical for preserving the fluidity and structural integrity of cellular membranes, but their susceptibility to peroxidation renders them a major vulnerability when iron-catalyzed oxidative stress is induced. Initially, PUFAs are esterified into PUFA-CoA by acyl-CoA synthetase long-chain family member 4 (ACSL4). Subsequently, the activated lipid intermediates are incorporated into phospholipid PUFAs via esterification with phosphatidylcholine by lysophosphatidylcholine acyltransferase 3 (Do and Xu, 2023; Stockwell, 2022). Lipoxygenase proteins (LOX), such as LOX-15 and LOX-12, catalyze the peroxidation of these phospholipid PUFAs, primarily at the plasma membrane and endoplasmic reticulum, leading to the formation of lipid hydroperoxides (Yang et al., 2016). Acyl-CoA synthetase long-chain family member 4 is considered a key ferroptosis-related enzyme, whereas LOX proteins have been identified as targets of ferroptosis inducers.

In addition, ROS contribute to this process. ROS interact with PUFAs to generate lipid-derived free radicals, thereby inducing membrane damage and promoting ferroptosis. The Fenton reaction, catalyzed by iron, further facilitates the production of these free radicals (D'Herde and Krysko, 2017). For instance, cancer cells treated with the ferroptosis inducer erastin undergo cell death as a result of iron-dependent lipid peroxidation (Ma et al., 2025). Furthermore, MDM2 and MDMX, which are negative regulators of P53, have been shown to promote ferroptosis via lipid metabolism mediated by peroxisome proliferator-activated receptor alpha (Venkatesh et al., 2020). Prospectively, modulation of ferroptosis may be achieved by targeting specific enzymes within this pathway, either to promote cell death in cancer cells or to prevent excessive damage in normal cells.

### 2.3 Amino acid metabolism

Amino acid metabolism is essential in biological systems and is closely linked to the regulation of ferroptosis (Angeli et al., 2017). Glutathione, a water-soluble tripeptide composed of *γ*-l-glutamyl-l-cysteinylglycine, exists in reduced (GSH) and oxidized (GSSG) forms. GSH acts as a crucial antioxidant by reducing H2O2 to H2O, scavenging free radicals, and functioning as a cofactor for glutathione peroxidase 4 (GPX4). Lipid hydroperoxides (LOOH) are reduced by GPX4 to non-toxic lipid alcohols, thereby preventing ferroptosis, an iron-dependent form of neuronal cell death (Bayır et al., 2020). However, glutathione depletion results in GPX4 inactivation, which subsequently increases lipid peroxidation and ultimately induces ferroptosis (Zhang et al., 2022).

The cystine/glutamate antiporter (System Xc-) plays a critical role in maintaining glutathione homeostasis. The system consists of a heterodimer composed of the light chain xCT (SLC7A11) and the heavy chain 4F2 (SLC3A2), which mediates a 1:1 exchange between intracellular glutamate and extracellular cystine. This exchange ensures intracellular cysteine availability, thereby promoting glutathione synthesis (Endale et al., 2023). An excess of extracellular glutamate impairs the function of this antiporter, thereby disrupting cysteine uptake and resulting in oxidative stress and ferroptotic cell death. Furthermore, inhibition of glutamine metabolism or cysteine deficiency increases ROS production and lipid peroxidation, thereby further facilitating ferroptosis (Galluzzi et al., 2015). Within the central nervous system, glutamate-induced neurotoxicity is associated with iron dysregulation, and both iron chelators and ferroptosis inhibitors have been demonstrated to alleviate this toxicity (Dixon et al., 2012). Due to its involvement in glutathione synthesis, System Xc- has been identified as a potential therapeutic target for diseases associated with iron-induced oxidative stress. Moreover, the association among glutamine metabolism, cysteine availability, and iron-driven pathology suggests that dietary or pharmacological modulation of amino acid metabolism could offer therapeutic benefits.

### 2.4 Dysregulation of mitochondrial metabolism

Mitochondrial dysfunction serves as a critical contributor to the mechanisms underlying ferroptosis. Excessive ROS production by mitochondria, which directly damages lipids, proteins, and nucleic acids, is capable of triggering multiple pathological signaling pathways. These pathways include mitochondria-dependent apoptosis, DNA damage, and ferroptosis. The voltage-dependent anion channel (VDAC), a membrane pore protein, is predominantly found on the outer mitochondrial membrane. VDAC performs metabolic and energy-producing functions, regulating mitochondrial activity and participating in cell survival and death signaling. VDAC is a transmembrane channel that facilitates the transport of ions and metabolites, including iron ions, across eukaryotic cell membranes (Mazure, 2017).

**Figure 1:**
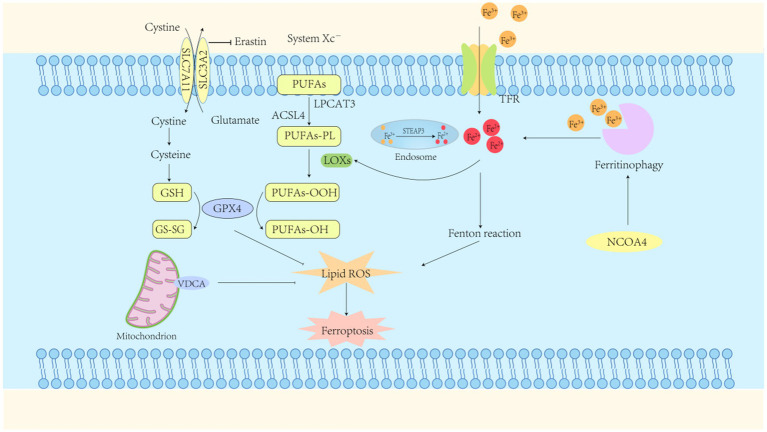
Ferroptosis mechanisms. Ferroptosis represents a form of regulated cell death characterized by iron-dependent lipid peroxidation. The primary mechanism driving this process is the accumulation of ferrous ions, which, in conjunction with reactive oxygen species (ROS), induce lipid peroxidation. A reduction in intracellular Glutathione peroxidase-4 (GPX4) activity impairs the GPX4-mediated metabolism of lipid peroxides. Ferrous ions subsequently catalyze the enhanced production of ROS from lipids, ultimately resulting in cell membrane disruption and subsequent cell death. GPX4, glutathione peroxidase-4; GSH, glutathione (reduced form); GS-SG, glutathione disulfide (oxidized form); TFR, transferrin receptor; System Xc-, cystine/glutamate transporter protein; NCOA4, nuclear receptor coactivator 4; ACSL4, long-chain acyl-CoA synthetase family member 4.

Inducers of iron-dependent cell death interact with VDAC2 and VDAC3 on the outer mitochondrial membrane, leading to changes in mitochondrial permeability. This interaction decreases the channel’s sensitivity to iron ions, restricts the efflux of molecules from mitochondria, and induces mitochondrial dysfunction. The subsequent release of oxidized metabolites further impairs cellular metabolism by inhibiting glycolysis, ultimately accelerating the progression of ferroptosis (Yagoda et al., 2007). Upon the application of the iron-dependent death inducer erastin to VDAC, the permeability of the outer mitochondrial membrane increases, resulting in the opening of ion channels. This disruption of intracellular homeostasis results in mitochondrial metabolic and oxidative dysfunction. As a result, an increase in reactive ROS production and enhanced lipid peroxidation occurs, ultimately culminating in cellular iron-dependent death (Chen et al., 2018). Cysteine deficiency, a critical component of GSH, leads to mitochondrial membrane hyperpolarization and the accumulation of LOOH, thereby triggering iron-dependent cell death. Thus, mitochondria are pivotal in regulating apoptosis mediated by iron and induced by cysteine deficiency (Gao et al., 2019) (Figure 1).

## 3 Ferroptosis and VaD

Ferroptosis is a complex form of cell death regulated by various cellular metabolic pathways. Its primary mechanism involves iron-dependent lipid peroxidation and ROS accumulation (Jiang et al., 2021), accompanied by GSH depletion, thereby impairing normal cellular function. Excessive lipid peroxidation disrupts membrane fluidity, permeability, and cellular integrity, ultimately resulting in cell death (Ji et al., 2022). Lipid peroxidation is a chain reaction catalyzed synergistically by iron and oxygen. When intracellular ROS accumulation exceeds the scavenging capacity of antioxidant substances, macromolecules such as phospholipids, enzymes, and PUFAs in the biofilm are catalyzed by LOX to form toxic LOOH. It not only alters the fluidity and permeability of the cell membrane but also induces oxidative damage to lipids and other macromolecules in the mitochondria. Under various cellular stress conditions, the mitochondrial membrane permeability transition pore transiently opens, resulting in the collapse of the mitochondrial transmembrane potential. This triggers a cascade reaction, releasing cytochrome c (Cyt c) into the cytoplasm and activating caspase-3, which may ultimately result in neuronal cell death (Riedl and Salvesen, 2007; Ott et al., 2002).

**Figure 2:**
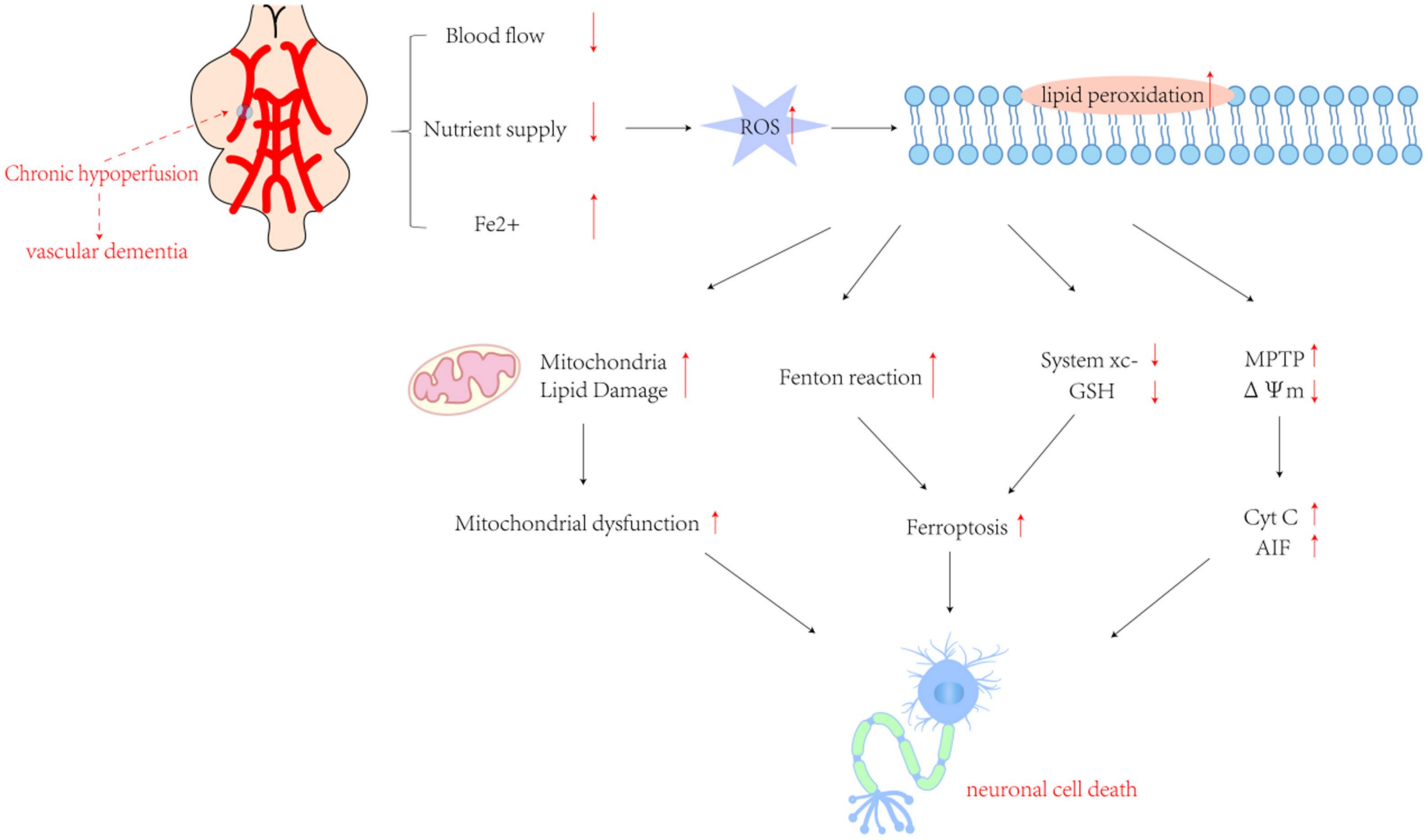
The cascade of pathological mechanisms triggered by chronic oxygen insufficiency in the brain involves a complex interplay of molecular events. Excessive production of reactive oxygen species (ROS) following cerebral ischemia and hypoxia initiates lipid peroxidation, setting off a series of downstream pathological processes. These factors encompass an intensified Fenton reaction, inhibition of Cystine/glutamate transporter protein (System Xc−) leading to ferroptosis, mitochondrial dysfunction caused by lipid damage, activation of inflammatory responses by pro-inflammatory factors, and mitochondrial collapse resulting from transient openings of the membrane permeability transition pore (MPTP) under stress. This collapse leads to the release of cytochrome c (Cyt c) and other pro-apoptotic molecules. Collectively, these processes trigger the apoptotic cascade. GSH, glutathione; ΔΨm, mitochondrial transmembrane potential; Cyt c, cytochrome c. AIF, apoptosis-inducing factor.

VaD can be triggered by CCH and reduced cerebral blood flow, leading to cerebrovascular lesions or impaired blood supply, ultimately resulting in blood vessel rupture and erythrocyte cleavage. This subsequently contributes to the intracellular accumulation of ferric ions, along with the generation of large amounts of ROS. ROS attack lipid molecules, initiating a lipid peroxidation reaction that ultimately results in ferroptosis in nerve cells (Figure 2). Mechanistic studies of neurodegenerative diseases, such as VaD and AD, have shown that pathological processes, including neuronal damage and synaptic loss, are strongly associated with ferroptosis (Adeniyi et al., 2023; Ou et al., 2022). Previous studies have reported significant iron ion accumulation and neuronal death in the CA1 region of rat models of VaD (Du et al., 2018). Recent studies have demonstrated that ferroptosis is a key mechanism underlying brain tissue damage and cognitive dysfunction following VaD (Fu et al., 2023; Ma et al., 2025). Given the critical role of iron as a redox-active element in cellular homeostasis, exploring the modulation of ferroptosis as an endogenous protective response to VaD may offer insights into strategies for enhancing its beneficial effects and mitigating the deleterious outcomes associated with vascular injury.

To mitigate the damage caused by ferroptosis in various neuronal cells, the responses of the iron detoxification and antioxidant systems may function as important endogenous protective mechanisms in VaD. Following VaD, cells initiate antioxidant defenses and activate enzymes such as HO-1 and superoxide dismutase, which help mitigate the effects of oxidative stress. It has been demonstrated that the inhibition of ROS can exert a protective effect and attenuate cognitive dysfunction by enhancing enzymatic defenses (Luca et al., 2015; Tan et al., 2025). Therefore, signaling pathways that promote antioxidant defense, such as the HO-1 signaling cascade, may represent novel targets for therapeutic strategies against VaD.

## 4 Classical and non-classical effects of HO-1

### 4.1 An overview of HO-1

Heme oxygenase (HO) is recognized as a key enzyme in heme metabolism and plays a vital role in enabling the body to cope with stress and injury. The breakdown of heme into biliverdin, carbon monoxide, and ferrous ions mediates these effects, exhibiting antioxidant and anti-inflammatory properties. To date, three HO isozymes (HO-1, HO-2, and HO-3) have been identified in mammals. HO-2 and HO-3 are the constitutive isoforms responsible for the majority of HO activity, whereas HO-1 expression is primarily induced by its physiological substrate, heme, or various external stimuli (Ryter et al., 2006). HO-1, commonly referred to as the 32 kDa heat shock protein, is encoded by the HMOX1 gene, which is localized to human chromosome 22q13.1 and has a molecular weight of 32,800 Da (Kutty et al., 1994). The protein encoded by this gene consists of 288 amino acids and includes four introns and five exons. The 5′ non-coding region of HMOX1 includes regulatory sequences such as the heme response element, metal response element, hypoxia response element, heat shock response element, antioxidant response element, as well as binding sites for activator protein-1 and specificity protein-1. These elements and binding sites are activated by various harmful stimuli, including heavy metals, cytokines, inflammation, ischemia, heat shock, hypoxia, and oxidative stress.

HMOX1 is distinguished by a “heme-binding pocket” that is oriented toward the cytoplasmic lysate and includes a histidine imidazole moiety for binding to heme iron. It is anchored to the endoplasmic reticulum (ER) membrane via a hydrophobic amino acid sequence at its C-terminal end (Ayer et al., 2016). In the promoter region of the HO-1 gene, a (GT) n dinucleotide repeat sequence is present and inversely affects the transcriptional activity of HO-1. Specifically, longer sequences are associated with reduced HO-1 activity, thereby weakening its antioxidant protection of cells (Cobley et al., 2018).

### 4.2 Classical effects of HO-1

HO-1 binds heme to a specific region of its active site, forming an enzyme-substrate complex that reduces ferric heme iron to its ferrous state, with electrons provided by nicotinamide adenine dinucleotide phosphate (NADPH) Cyt c (P450) reductase. Molecular oxygen binds to the complex, forming a transient oxygen species. Oxygen bound to iron is converted into a hydroperoxide intermediate (Fe^3+^-OOH), with the terminal oxygen of Fe^3+^-OOH subsequently attacking the α-carbon of the porphyrin ring, forming iron α-hydroxyheme. This intermediate reacts with molecular oxygen, forming the ferrous verdoheme-HO complex and producing carbon monoxide (CO). The conversion of heme to bilirubin-iron chelate requires the involvement of oxygen and the supply of reducing equivalents. The bilirubin-iron complex is subsequently reduced back to the ferrous state by reductase activity. In the next phase of the HO reaction, the resulting bilirubin (BV) is further converted into bilirubin-Ixα, catalyzed by bilirubin reductase (Kikuchi et al., 2005).

HO-1 is the primary endogenous source of CO, which acts as a key second messenger in the central nervous system (CNS). Over time, various roles of CO have been identified in human systems, including activation of the p38-MAPK signaling pathway, reduction of endothelial cell apoptosis, and induction of anti-inflammatory effects. In the brain, CO activates soluble guanylate cyclase, mediating neurotransmitter effects and inducing vasodilation (Verma et al., 1993). Under oxidative stress and inflammatory conditions, the interaction between CO and Cyt c oxidase (COX) disrupts the mitochondrial redox balance and contributes to the regulation of mitochondrial quality control (Gasier et al., 2022). The upregulation of HO-1 expression or administration of CO triggers potent anti-inflammatory responses in monocytes and macrophages, reduces tissue damage, and influences their involvement in initiating immune responses (Bell and Maines, 1988). Numerous studies have also shown that CO produced during HO-1 catabolism influences the expression and transcription of GnRH genes. Carbon monoxide diffuses across the retina into the venous circulation, traveling through bodily fluids to the brain, where it mitigates neuroinflammation and exerts protective effects (Nowak et al., 2022).

**Figure 3:**
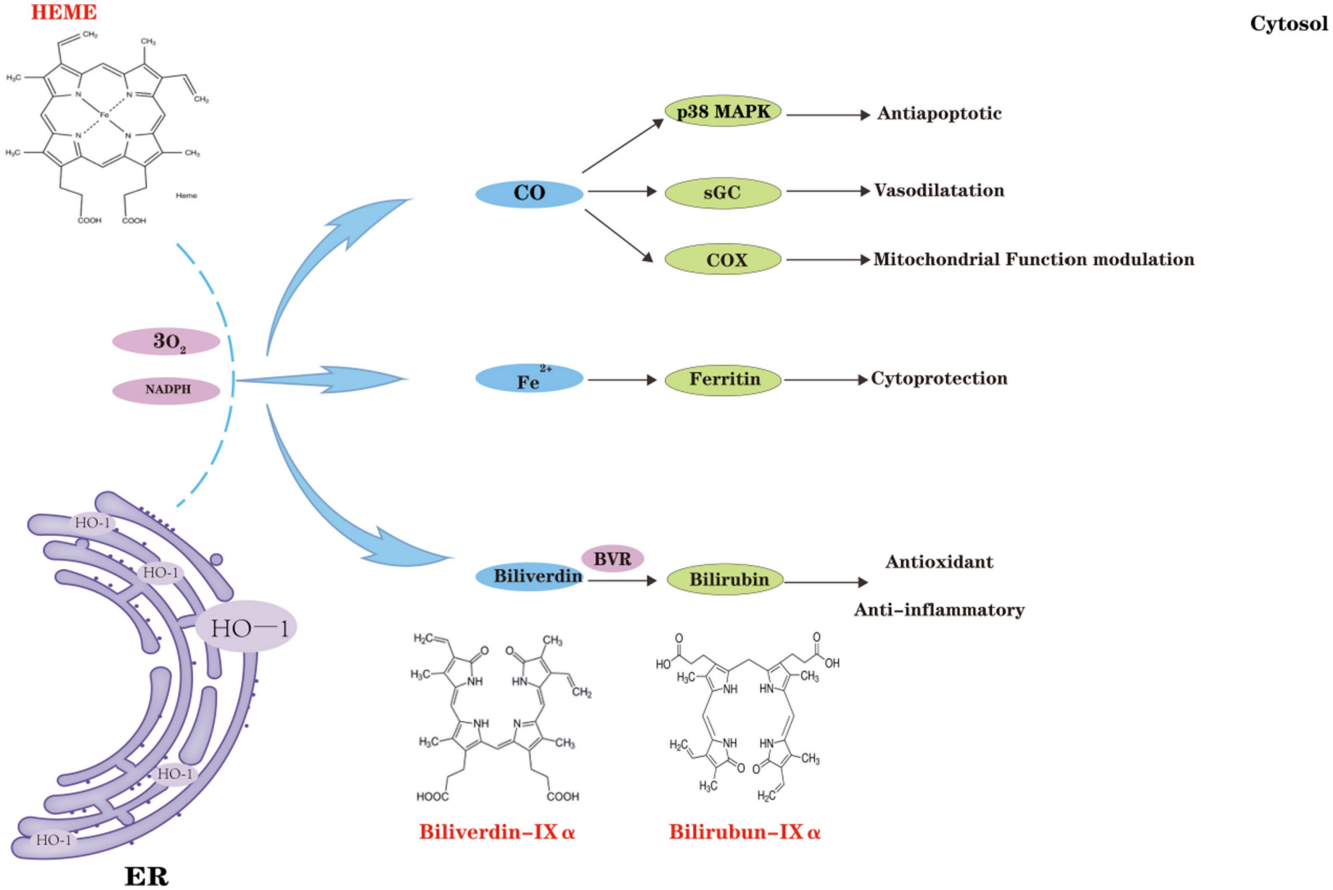
Classical effects of heme oxygenase-1 (HO-1). HO-1 catalyzes the degradation of hemoglobin, leading to the production of biliverdin (BV), carbon monoxide (CO), and ferric iron (Fe^3+^). CO, produced by HO-1, regulates cellular anti-apoptotic pathways, including the p38 MAPK pathway, and activates soluble guanylate cyclase (sGC), which functions as both a neurotransmitter and a vasodilator in the brain. Under oxidative stress and inflammatory conditions, CO, in cooperation with Cyt c oxidase (COX), affects mitochondrial redox homeostasis and orchestrates mitochondrial quality control. Furthermore, ferrous iron (Fe^2+^) generated by HO-1 induces ferritin expression, thereby conferring cellular protection. BV, along with its reduced form BR, exhibits potent anti-inflammatory and antioxidant properties, further enhancing cellular protection.

The BV and bilirubin (BR), produced by HO-1 as reducing agents, function as potent antioxidants and anti-inflammatory mediators, displaying cytoprotective properties against harmful stimuli in both *in vivo* and *in vitro* contexts. BV attenuates pro-inflammatory responses and inhibits toll-like receptor 4 (TLR4) signaling by modulating the nuclear factor κB (NF-κB) pathway, a key factor in the pathogenesis of neurological disorders. Conversely, BR has been extensively shown to act as a free radical scavenger at micromolar concentrations *in vitro* and inhibit adhesion molecule signaling (Stocker et al., 1987; Mazzone et al., 2009; Vogel and Zucker, 2016). The final product of the HO-1 reaction, Fe^2+^, exhibits toxicity primarily due to its chemical properties, which enable it to donate electrons, leading to the formation of hydroxyl radicals via the Fenton reaction. Elevated Fe^2+^ levels, mediated by iron transport pumps, stimulate ferritin expression, which binds iron and exhibits anti-apoptotic properties (Ferris et al., 1999; Berberat et al., 2003) (Figure 3). Ferritin binds free Fe^2+^, thereby limiting free radical generation. In contrast, unbound Fe^2+^ can participate in the Fenton reaction, leading to the production of reactive oxygen species (Miotto et al., 2020). HO-1-mediated production of CO, BV, and BR may reduce cellular damage by counteracting oxidative stress and inflammation. However, Fe^2+^ toxicity must also be addressed when designing HO-1-based therapies. Future research should aim to balance the protective and harmful effects of HO-1 to maximize its therapeutic value.

### 4.3 Non-classical effects of HO-1

Recent studies have demonstrated that, in addition to its enzymatic function, HO-1 performs several “non-canonical” roles, including protein–protein interactions, subcellular localization (e.g., secretion into the extracellular space), and regulation of cellular metabolism (Vanella et al., 2016; Barone and Butterfield, 2015). Protein–protein interactions among HO isoforms were first described in 1977; Weng et al. (2003) elucidated the interaction mechanism between HO-1 and HO-2, further confirming the effect of the HO-1/HO-2 protein complex on HO activity. This interaction modulates HO activity in specific tissues when the two coenzymes are co-localized, a process that may be critical for the cytoprotective effects of HO in brain tissue.

Initially discovered in the endoplasmic reticulum, HO-1 has also been detected in mitochondria, nuclei, and follicles in subsequent studies. The mitochondrial localization of HO-1 plays a critical role in the metabolism of heme proteins within the mitochondria and significantly contributes to protection against related pathophysiological conditions, including neurodegenerative diseases. Under stress conditions, active HO-1 in the mitochondria regulates heme availability for mitochondrial cytochromes (Converso et al., 2006). A significant non-classical function of HO-1 is its nuclear translocation. Bioinformatics analyses have identified a nuclear import signal sequence in HO-1, facilitating its translocation into the nucleus under hypoxic or stress conditions (Luo et al., 2021; Yang and Wang, 2022; Biswas et al., 2014). This translocation affects nuclear function and results in reduced HO activity. Furthermore, excitotoxic damage has been shown to significantly elevate HO-1 protein expression in primary astrocyte cultures, accompanied by translocation of the protein to the nucleus (Li et al., 2004). Lin et al. (2007) observed that HO-1 translocates to the nucleus under hypoxic conditions. This phenomenon is closely associated with enhanced activation of antioxidant-responsive promoters and transcription factors, particularly the well-known HO-1 regulators AP-1 and NFκB (Lavrovsky et al., 1993; Lavrovsky et al., 1994). Furthermore, docking studies have shown (Vanella et al., 2016) that HO-1 may interact with the p65 subunit of NFκB. However, the interaction surface of p65 does not appear to include the DNA-binding domain, suggesting that inhibition may occur through allosteric modulation. Recent studies have suggested a link between HO-1 and the regulation of G-tetrasomes (G4). G4 is a non-canonical DNA structure formed by guanine-rich sequences, consisting of two or more stacked G-quadruplexes (Davis, 2004). Heme serves as a stabilizing ligand for the G4 structure, enhancing its stability and maintaining the normal concentration of free heme in the nucleus (Gray et al., 2019). Although studies have shown that HO-1 co-localizes with G4 in the nucleus (within 40 nm), as confirmed by the proximity ligation assay (PLA), no direct evidence of a binding interaction has been provided (Krzeptowski et al., 2021). Notably, the nuclear localization of HO-1 facilitates the clearance of G4, thereby enhancing its functional role within the nucleus (Chudy et al., 2024).

**Figure 4:**
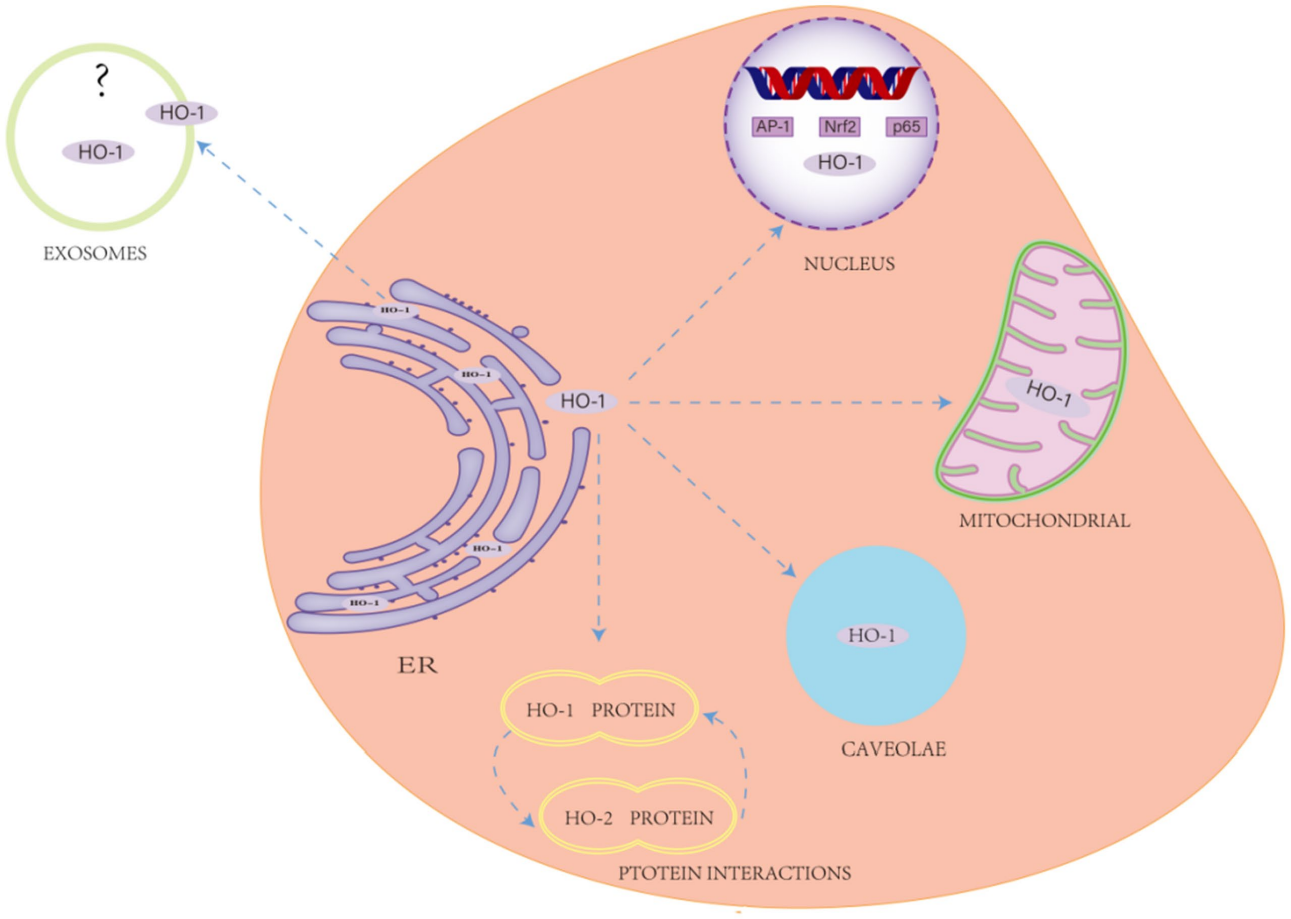
Non-classical effects of heme oxygenase-1 (HO-1). This encompasses protein–protein interactions, subcellular localization (e.g., HO-1 is primarily found in the endoplasmic reticulum (ER), with additional isoforms detected in mitochondria, the nucleus, exosomes, and vesicles), and the regulation of cellular metabolism.

Additionally, HO-1 has been shown to be secreted into extracellular spaces (Figure 4). Research has shown (Mueller et al., 2010) that serum levels of HO-1 are elevated in individuals with Alzheimer’s disease, positively correlating with the degree of cognitive decline. Schipper et al. (2000) reported a decrease in HO-1 levels in the cerebrospinal fluid of individuals with Alzheimer’s disease. The functional role of HO-1 in extracellular fluid, however, remains poorly understood. These observations imply that various HO isoforms, especially HO-1, might affect brain function through non-canonical pathways and could potentially serve as biomarkers for these disorders.

## 5 Role of HO-1 in ferroptosis associated with VaD

### 5.1 Regulation of key signaling pathways

In VaD, HO-1 plays a critical role in enhancing neuroprotection and regulating ferroptosis through the modulation of several key signaling pathways. These signaling pathways are crucial for combating oxidative stress, modulating inflammatory responses, and regulating mitochondrial dysfunction and cell survival (Bauer et al., 2024). Biosignaling molecules closely associated with HO-1 expression and function include both its upstream signals [e.g., Nrf2 (nuclear factor erythroid 2-related factor 2), MAPK, STAT3] and its downstream signals [e.g., peroxisome proliferator-activated receptor-γ coactivator-1 (PGC-1), estrogen-related receptor, and hypoxia-inducible factor-1 (HIF-1)]. For example, Nrf2, an important transcription factor *in vivo*, participates in the body’s antioxidant stress response by regulating the metabolism of GSH, iron, and lipids, as well as mitochondrial function, and activates the expression of several target genes involved in regulating ferroptosis (Shen et al., 2017). In addition, Nrf2 can influence the expression of genes encoding GSH-synthesizing proteins, such as SLC7A11, GCLC/GLCM, and GSS (Saha et al., 2023), thereby inhibiting peroxide production. HO-1, a target gene of Nrf2, exerts a critical protective effect against brain injury. Recent studies have demonstrated that the Nrf2/HO-1 pathway enhances the brain tissue’s tolerance to ischemic oxidative damage (Liao et al., 2023; Yadav et al., 2018). Additionally, this pathway can inhibit activated protein kinase C and reduce coenzyme II (nicotinamide adenine dinucleotide phosphate, NADPH) activity, alleviate ER stress and lipid accumulation (Jin et al., 2021), scavenge accumulated oxidative products, and reverse oxidative stress damage in nerve cells, thereby delaying or preventing the progression of VaD. Wang et al. (2023) demonstrated that fosetyl sanshin (FSS) significantly inhibited lipid peroxidation and ferroptosis in a VaD mouse model through pharmacological activation of the Nrf2/HO-1 pathway. Li et al. (2022) found that Aspergillus (GAS) activated the Nrf2/HO-1 pathway by upregulating HO-1 expression, which enhanced antioxidant capacity, reduced free iron accumulation, and ultimately inhibited ferroptosis in a rat model of VaD. This finding indirectly suggests that the Nrf2/HO-1 pathway is a key negative regulator of ferroptosis in neuronal cells in VaD.

### 5.2 Antioxidant stress response

Oxidative stress is induced by the excessive production of ROS and reactive nitrogen species (RNS), resulting in extensive damage to cellular structures (Patel, 2016). When oxidative stress occurs in neuronal cells, the levels of free radicals increase, and HO-1 is rapidly activated. HO-1 expression is regulated and repressed by the transcription factor Bach1 (BTB and CNC homology 1) and activated through the regulation of Nrf2 (Dun et al., 2021). Upstream of the transcription start site of the HMOX1 promoter, two key enhancer regions are located at −4 kb (E1) and −10 kb (E2). These regions contain multiple antioxidant response elements (AREs), which serve as binding sites for both Nrf2 and the transcriptional repressor Bach1. Under normal physiological conditions, Bach1 binds to AREs in the HO-1 promoter, thereby repressing HO-1 gene expression. However, under oxidative stress, Bach1 dissociates from the HO-1 promoter, and stress signals induce the dissociation of Nrf2 from Kelch-like ECH-associated protein 1 (Keap1), which then binds to AREs and initiates the downstream expression of oxidative stress-resistant HO-1 to prevent ROS-induced cellular damage and maintain the organism’s antioxidant response (Zhang et al., 2019).

Activation of HO-1 promotes the expression of the System Xc- system and accelerates cystine/glutamate transport, leading to the scavenging of accumulated lipid peroxides and the inhibition of oxidative stress, thereby reducing cellular sensitivity to ferroptosis. The production of CO, Fe^2+^, and BR is also promoted, which are products that protect neuronal cells from ischemic and hypoxic damage, maintain iron homeostasis, and reverse the progression of VaD through multiple pathways (Chiang et al., 2021). As a key cell signaling molecule, CO not only regulates vascular tone but also confers resistance to ER stress in endothelial cells by down-regulating CCAAT/enhancer-binding protein homologous protein expression and up-regulating the Nrf2/HO-1 pathway (Yang et al., 2014). Free iron binds to proteins to form ferritin, which scavenges oxygen radicals; this chelation reverses the oxidative process in cells by reducing free iron, thereby inhibiting ferroptosis (Orino et al., 2001). As a potent endogenous antioxidant, BR not only effectively scavenges superoxide and helps cells partially resist oxidative stress, but also possesses significant iron chelating ability, which effectively chelates Fe^2+^ and Fe^3+^, alters the distribution of intracellular iron, and reduces the cellular uptake of iron, thus constituting an important part of the endogenous antioxidant defense system (Llido et al., 2023).

### 5.3 Regulation of neuroinflammation

The occurrence of ferroptosis is closely associated with inflammation, and ROS produced during ferroptosis can activate microglial cells, prompting the release of inflammatory factors and exacerbating neuroinflammation (Yeerlan et al., 2024). Meanwhile, damage-associated molecular patterns are produced during ferroptosis, and these molecules cause glial activation through the activation of neuroimmune pathways, leading to the overproduction of inflammatory factors (Mohan et al., 2024). Following VaD, inflammatory cells are rapidly activated, releasing large amounts of ROS and pro-inflammatory mediators, including inflammatory cytokines and chemokines. These mediators exacerbate blood–brain barrier disruption, triggering an inflammatory cascade that results in neuronal death. By upregulating HO-1 expression, the entry of Nrf2 into the nucleus is inhibited, and the activation of NF-κB and tumor necrosis factor α (TNF-α) are reduced, thus effectively reducing the inflammatory response (O'Rourke et al., 2024; Feng et al., 2024). NF-κB is a key transcription factor that regulates the inflammatory response. Upon its activation, NF-κB translocates to the nucleus and induces the expression of various pro-inflammatory genes, including TNF-α, IL-1β, and IL-6 (Cornice et al., 2024; Zhong et al., 2024).

Neuroinflammation involves all cells of the CNS, with microglial activation playing a pivotal role in CNS injury (Long et al., 2024). Neuroinflammation and ferroptosis resulting from cerebrovascular injury can be attenuated by modulating the microglial phenotype. In the later stages of cerebrovascular disease, HO-1 is believed to exert a protective effect by activating microglia and astrocytes. For example, Chen et al. (2025) demonstrated in an intracerebral hemorrhage (ICH) model in mice that HO-1 activates microglia in the later stages of ICH, promotes phagocytosis, enhances the absorption of hematomas and cellular debris, induces neurogenesis and angiogenesis, and thus facilitates brain tissue recovery while attenuating secondary brain injury. Chen-Roetling and Regan (2017), on the other hand, demonstrated that selective overexpression of HO-1 in astrocytes attenuates blood–brain barrier disruption, reduces perihematoma cell injury, improves neurological deficits, and lowers mortality in an ICH model using rat autologous arterial blood. This mechanism of brain cell protection by astrocytes is closely linked to the trophic support they provide to neurons, and HO-1 enhances this support.

### 5.4 Regulation of mitochondrial dysfunction

Mitochondria, as subcellular organelles containing DNA, provide energy to cells through oxidative phosphorylation (Antolínez-Fernández et al., 2024) and are implicated in various pathophysiological processes in VaD. In VaD, an insufficient oxygen supply to the brain, reduced blood flow, and decreased intracellular oxygen partial pressure result in mitochondrial oxidative phosphorylation dysfunction, inhibition of protein synthesis, insufficient ATP production, and energy depletion, ultimately leading to sodium-potassium pump failure, calcium overloading, and disturbances in cytosolic ions (e.g., Ca^2+^, Na^+^, H^+^, ROS), which subsequently increase. These ROS generate hydroxyl radicals via the Fenton reaction, which attack intracellular proteins, DNA, and lipid membranes, thereby disrupting mitochondrial function and cellular integrity, and promoting the occurrence of ferroptosis. Therefore, the maintenance of mitochondrial homeostasis may represent a potential therapeutic strategy for preserving normal neuronal function in VaD.

Induction of HO-1 results in the release of CO, which regulates the activity of transcription factors such as NRF-1 and PGC-1α through the Akt, AMPK, MAPK, and Sirt1 pathways, thereby promoting the expression of these transcription factors and the generation of antioxidant enzymes involved in mitochondrial biosynthesis. Phosphorylation of Akt, AMPK, and MAPK enhances PGC-1α activity, promoting its cytosolic translocation and initiating mitochondrial biogenesis transcriptional programs. The PGC-1 protein is a key regulator of mitochondrial biogenesis and energy production, promoting the transcription and replication of the mitochondrial genome by activating downstream nuclear respiratory factor (NRF1) and mitochondrial transcription factor A (TFAM), thereby improving mitochondrial function (LeBleu et al., 2014). The HO-1/PGC-1α pathway not only protects cells from oxidative stress by strengthening the antioxidant defense system but also provides mitochondrial protection by regulating mitochondrial autophagy and inducing mitochondrial biosynthesis.

In addition, HO-1, as an endoplasmic reticulum-resident protein, can be translocated to the mitochondria, where it stabilizes mitochondrial function under stress. Recently, it has been found that physiological doses of CO, produced during the HO-1 enzymatic reaction, can act as intracellular second messengers, promoting mitochondrial regeneration and restoring mitochondrial membrane potential, thereby reducing mitochondrial ROS production (Huang et al., 2018). However, its specific relationship with ferroptosis remains to be further elucidated.

### 5.5 Divergent roles of HO-1 in ferroptosis: between protection and promotion

Although numerous studies have suggested that HO-1 may exert neuroprotective effects in VaD by regulating ferroptosis, an in-depth analysis of the existing literature reveals that the findings are not entirely consistent. For instance, moderate induction of HO-1 has been shown to reduce oxidative stress and inflammation, thereby protecting neurons (Wang et al., 2023; Ma et al., 2025), whereas overexpression of HO-1 has also been associated with iron accumulation and lipid peroxidation, potentially exacerbating ferroptosis and further impairing neurological function (Fu et al., 2023).

These discrepancies may stem from variations in experimental design, including animal species or cell lines, types and dosages of HO-1 inducers, treatment duration, and the use of chelating agents or antioxidants. In addition, heterogeneity may be further introduced by the use of different assays for ferroptosis (e.g., GPX4 activity, levels of lipid peroxidation, MDA assays) and by variations in endpoint definitions.

In this review, the dual role of HO-1 was analyzed, highlighting its “double-edged sword” effect in ferroptosis, which may exert neuroprotective effects or, under certain conditions, promote cell death. It is suggested that this dual action of HO-1 is not random but rather context-dependent. Its overall effect is likely influenced by multiple factors, including cellular iron buffering capacity, mitochondrial function, and antioxidant homeostasis. Therefore, future studies should aim to define the threshold at which HO-1 shifts from protective to deleterious roles and promote the standardization of experimental protocols to facilitate cross-study comparison and data integration.

## 6 Conclusion and future perspectives

HO-1 is thought to exert beneficial anti-oxidative stress effects in the regulation of VaD processes triggered by ferroptosis, thereby mitigating and delaying brain damage caused by oxidative stress. The beneficial functions of its enzymatic activity and reaction products are primarily involved in the regulation of signaling pathways, oxidative stress, inflammation, and mitochondrial dysfunction. HO-1 is also suggested to play compartment-specific roles, potentially exhibiting activity-dependent functions in vesicles and mitochondria. In addition, the Nrf2/HO-1 and HO-1/PGC-1α pathways are recognized as critical components of intracellular signaling cascades involved in neurogenesis and mitochondrial biogenesis. Several drugs, including arginine and heme-arginine esters, as well as Hmox1 gene therapy approaches (Andreas et al., 2018; Singh et al., 2020), are currently employed to enhance HO-1 expression. Preclinical studies, including gene therapy approaches by Abraham et al. (2007) and pharmacological interventions using CO-releasing molecules as described by Ling et al. (2018), have demonstrated that enhancing HO-1 expression or mimicking its enzymatic products holds therapeutic potential for VaD. Most notably, CO, the end product of HO-1 activity, has been suggested as a therapeutic mimic of HO-1, either through gas administration or pharmacological delivery of chemical donor compounds, including transition metals, CO-releasing molecules (CORMs), and organic CO donors containing carbon monoxide-releasing molecules (Steiger et al., 2017; Lazarus et al., 2020; Yang et al., 2020). Although HO-1 is commonly associated with cytoprotection and inhibition of cell death processes, excessive activation of HO-1 releases large amounts of Fe^2+^, leading to the accumulation of unstable iron pools and corresponding deleterious cyclic effects that exacerbate oxidative stress, presenting a dual effect that characterizes HO-1 as a double-edged sword.

While several key pathways of HO-1 regulation in ferroptosis have been identified, the precise molecular switches that determine its protective versus detrimental effects remain incompletely understood. Moderate HO-1 induction provides neuroprotection, excessive upregulation may paradoxically accelerate ferroptosis through iron overload, highlighting the need for precise therapeutic modulation. To better understand the mechanism of HO-1 action in VaD and its potential therapeutic value, future investigations should be directed toward the following objectives: (1) clarifying the specific mechanisms of HO-1-derived metabolites and comparing their effects across various disease models; (2) investigating the signaling pathways through which HO-1 modulates ferroptosis (e.g., the Nrf2/HO-1 axis) and their context-dependent roles in different pathological states; (3) conducting multicenter, double-blind, prospective clinical trials with large sample sizes to validate the efficacy and safety of HO-1-targeted therapeutic strategies in patients with VaD. In addition, future research should be directed toward developing effective therapeutic strategies, including the investigation of HO-1 modulators with balanced redox properties and the design of multimodal imaging tools for *in vivo* iron monitoring, which may offer novel approaches for the treatment of VaD.

## 7 Methods

In this study, we conducted a comprehensive literature search across several prominent databases, including PubMed, Embase (via OVID), Cochrane CENTRAL, CINAHL, and Web of Science, with data retrieval extending through December 2024. The search strategy was meticulously tailored to the unique characteristics of each database. Key search terms, including “Heme Oxygenase 1,” “Ferroptosis,” “Oxidative Stress,” “Lipid Peroxidation,” and “Vascular Dementia,” were employed to identify relevant studies. Literature screening and data extraction were performed independently by two investigators, in strict adherence to the inclusion and exclusion criteria outlined by Elsevier. Discrepancies between the investigators were resolved through collaborative discussion or third-party arbitration. The screening process commenced with an initial assessment of article titles, followed by a thorough review of abstracts and full texts to ensure the inclusion of studies pertinent to the research focus.

Inclusion criteria were established as follows: (1) original studies, including *in vitro* and *in vivo* experiments, that investigated the role of HO-1 in ferroptosis or VaD; (2) Clinical or preclinical studies involving interventions based on HO-1 or its active metabolites (e.g., CO, biliverdin, bilirubin); (3) studies specifically employing models of VaD or chronic cerebral hypoperfusion; (4) no restrictions were placed on the species, sex, age, or weight of experimental animals or cells; (5) only peer-reviewed original studies published in English were included.

The exclusion criteria comprised: (1) duplicate publications or studies derived from the same data source; (2) studies with unclear experimental design or a high risk of methodological bias; (3) studies with incomplete or inaccessible outcome data; (4) reviews, conference abstracts, editorial comments, non-peer-reviewed literature, and unpublished material were excluded.

This review was conducted as a narrative synthesis rather than a systematic review or meta-analysis, as the included studies focused on mechanistic aspects and exhibited considerable heterogeneity. These differences were addressed by assessing the consistency of results across various types of studies (e.g., *in vitro* and *in vivo*) and study designs. Discrepancies, when identified, were discussed in the relevant sections (see Section 5). Studies were selected based on relevance and methodological clarity, regardless of species, model type, or outcome measures. Due to substantial variability in study procedures and outcome measurements, a meta-analysis was deemed inappropriate at this stage.
